# The Treatment of Cerebral Hemorrhage

**Published:** 1897-12

**Authors:** 


### THE TREATMENT OE CEREBRAL HEMORRHAGE.

In *Treatment*, for July 8, Dr. Byrom Bramwell has an article on this subject, of which the following is the substance:

"At the beginning of an attack of cerebral hemorrhage, says the author, the first indication for treatment is to try to arrest the bleeding and limit the extravasation, and this is done by lessening the activity of the cerebral circulation. The head and shoulders should be raised rather than lowered. An ice-bag should be applied to the head and warmth to the feet. Leeches may be applied behind the ear, and a drop or two of croton oil administered. Venesection, bleeding from the temporal artery, compression of the common carotid artery, and ligaturing the carotid artery on the side of the hemorrhage are, he says, other methods which have been recommended.

"Bleeding, Dr. Bramwell believes, is useful and especially indicated in those cases in which the face, head and neck are turgid, the pulse is hard, full and slow, and the left ventricle is hypertrophied. It is contra-indicated, however, in cases in which the pulse is feeble, rapid or irregular, the heart dilated or weak, and the patient very old or debilitated.

"A brisk, watery purge acts, he says, in very much the same way as a moderate bleeding, but for the production of such a purge time is required; consequently, in many cases, venesection is preferable. In cases in which the advisability of bleeding is doubtful, a drop or two of croton oil and an enema may be administered. The practice of applying a blister to the nape of the neck is, Dr. Bramwell thinks, of doubtful advantage. In several cases, he says, it is useless, and in slight cases in which coma is speedily recovered from it is unnecessary. If a blister is to be applied at all, it is probably best applied to the shaved scalp between the ears over the top of the head. The ice-bag may be applied over the top of the blister.

"Internal remedies Dr. Bramwell considers doubtful, as they have not much influence in arresting the bleeding, although nitrate of amyl, or nitrate of sodium, is perhaps useful in some cases in which the pulse is hard and tense; venesection, however, he considers a better remedy.

"If coma becomes deeper and deeper, the pulse slower and slower, and the respiration more and more affected, and intracranial pressure is evidently steadily increasing as the result of gradually increasing hemorrhage, the advisability of trephining and tapping the hemorrhagic cavity, and so preventing rupture into the lateral ventricles—an event which is certainly and rapidly fatal—should be considered. Such cases are comparatively rarely met with.

"The second indication, continues the author, is to attend to the condition of the bladder, and to take means to prevent, if possible, the formation of a bed-sore. The patient should be placed at once, or as soon as he can be moved without risk, upon a water-bed. Care must be taken, too, that the hot bottles which are applied to the feet are not too hot. Owing to the comatose, or semi-comatose condition, the patient will not, of course, make any complaint (the nurse has, under such circumstance, to feel for him), and, owing to the diminished trophic resistance of the skin, a degree of heat which would not be prejudicial to a healthy person may easily blister and burn the skin of a patient suffering from cerebral hemorrhage. If there is retention of urine, the bladder should be empted by the catheter at regular intervals; if there is incontinence, the patient should be kept dry and clean. This is a most important point, says Dr. Bramwell, for the development of a bed-sore is one of the chief dangers in cases which do not immediately prove fatal.

"The third indication is to sustain the vital powers by appropriate feeding and, if necessary, by the administration of cardiac tonics and stimulants. It is important to avoid giving anything which is likely to produce vomiting, for the straining which attends the act of vomiting may reopen the ruptured vessel, or, if the bleeding is still going on, increase it. For the same reason stimulants should be withheld, unless they are absolutely required. If the heart is failing and the pulse rapidly running down, stimulants must, of course, be administered, even at the risk of increasing or re-exciting the hemorrhage.

"During the comatose state, Dr. Bramwell continues, the administration of food and liquids by the mouth requires to be conducted with great care and caution.

"A nutrient enema may be given every four hours, and, if necessary, it may be supplemented every now and again by a nutriment suppository. During the stage of coma, mucus, saliva, etc., are apt to accumulate in the mouth and pharynx, and add to the difficulty of the respiration and the tendency to death from asphyxia; for it must be remembered that in some cases the patient dies during the stage of coma from failure of the heart's action, in others from asphyxia and failure of the respiration, in others from the two conditions combined. In others again, death is preceded or attended by hyperpyrexia.

"By attention to posture (turning the patient on his side, turning the head to one side, etc.,), it is in many cases possible to avoid the accumulation of mucus, etc., at the back of the throat, and so to diminish the risk of asphyxia. The relief is, however, in most cases merely temporary. In cases of cerebral hemorrhage in which the conditions are developed, the result is almost always fatal. It is very different when we are dealing with the status epilepticus. In that condition, Dr. Bramwell says, he has undoubtedly, in more than one case, by preventing the accumulation of mucus, saliva, etc., in the back of the throat, and so preventing asphyxia, saved the life of the patient.

"Provided the patient can swallow, a teaspoonful or two of milk may from time to time be given by the mouth, but once the bowels have been thoroughly well opened it is better to feed the patient by the rectum. If there is difficulty in swallowing, if the administration of fluids by the mouth produces coughing or choking, the feeding should be entirely rectal. Alcoholic stimulants, digitalis, etc., may be given by the same channel, or strychnine (a drop or two of the liquor every two hours) may be administered hypodermically, the effect being, of course, carefully watched.

"Possibly in some cases in which the respiration is much embarrassed and death from asphyxia seems imminent, oxygen inhalations might be beneficial.

"The main objects of treatment during the first stage of cerebral hemorrhage are to arrest the bleeding and to tide the patient through the stage of coma.

"The fourth indication, continues the author, is to prevent and allay the secondary cerebral inflammation.

"When symptoms indicative of this (a rise in temperature, headache, muscular twitchings, rambling, a return of the coma, etc.,) develop a brisk purge may be again administered, cold (an ice-bag) reapplied to the head, and bromide of potassium and chloral hydrate given in addition to the iodide.

"If, during the stage of secondary cerebral inflammation, the pulse becomes very quick, feeble, or intermittent, cardiac stimulants—digitalis, strophanthus, strychnine, etc.—must be given; alcohol is probably better avoided. If the pulse tension is high, the administration of remedies which depress the force and violence of the heart's action, such as aconite or nitrate of sodium, may perhaps be employed with advantage in some cases in addition to purgation.

"As the symptoms of this secondary inflammation subside the use of bromide of potassium and chloral hydrate should be discontinued.

"After the symptoms indicative of this secondary inflammation pass off, complete rest must still be enjoined until the acute changes round the clot have subsided. The use of iodide of potassium, with perhaps a small dose of carbonate of ammonium or tincture of nux vomica, should be continued. During this, the early stage of convalescence, the patient must be carefully fed, the condition of the bladder and rectum attended to, and any cystitis or bed-sores which may have developed treated. At this stage of the case gentle massage is useful. Faradaism of the paralyzed muscles, strychnine, and too active attempts at voluntary movement of the paralyzed parts, all of which may be most useful a little later, should be avoided, or, if employed, administered with great caution.

"Some authorities, Dr. Branwell continues, recommend the application of the constant electric current to the head—one pole being placed just above either mastoid process. The constant current, by its atalytic action, is supposed to aid the absorption of inflammatory products and to promote the nutrition and restoration of the damaged nerve elements. It is very doubtful, he thinks, if electricity applied in this way is of any real use. If it is employed the greatest care should be taken to use a weak current, and the effects which the current produces on the patient should be carefully watched.

"In severe cases of hemiplegia the tendency to the development of contractures should be remembered, and passive movements (more especially of the fingers, wrist, and elbows, for it is at these parts that the contractures are most apt to be developed) carefully and diligently praticed.

"When there is reason to suppose that the acute changes have subsided—*i. e.*, at the end of six weeks or two months—the treatment appropriate for an ordinary case of chronic hemiplegia may be employed. A more liberal dietary may be allowed; the patient should be encouraged to practice systematic voluntary movements; general tonics, such as quinine and small doses of strychnine may be given internally, and massage and electricity judiciously and cautiously applied to the paralyzed muscles.

"The treatment (amount of exercise, etc.) must, of course, Dr. Branwell adds, be carefully and judiciously regulated in accordance with the conditions which are present in each individual patient (the severity of the paralysis, etc.), the state of his heart, arteries, kidneys, etc., being taken into account.

"Concerning the prevention of subsequent attacks, Dr. Branwell thinks that much can be done to prevent and defer a second rupture. All exciting causes should be avoided; it is especially necessary to reduce the blood pressure when the pulse tension is excessive, such as sudden efforts, mental excitement, sudden exposure to cold, straining at stool, etc. A patient who has had an attack of cerebral hemorrhage, however slight, should lead a quiet, routine life, and if his business entails much bodily exertion, mental strain or excitement, he should be advised to give it up. In some cases, however, it is usually preferable to allow the patient to continue his work in a modified way rather than to worry in his idleness. The risks entailed by the work and the risks entailed by the idleness and the want of occupation have to be weighed one against the other.

"The diet should be light and nutritious; if the patient is gouty, if his kidneys are cirrhotic, if his blood pressure is high, a non-nitrogenous diet is best. In these cases, says Dr. Branwell, alcohol should be prohibited; a certain amount of tobacco, however, may be allowed. A certain amount of gentle exercise is beneficial, but sudden exertions, running for trains, etc., should be rigidly avoided."—*N. Y. Medical Journal.*

